# Burden of Neural Tube Defects and Their Associated Factors in Africa: A Systematic Review and Meta-Analysis

**DOI:** 10.1155/2023/9635827

**Published:** 2023-06-21

**Authors:** Reta Wakoya, Mekbeb Afework

**Affiliations:** ^1^Department of Biomedical Science, Menelik II Medical and Health Science College, Addis Ababa, Ethiopia; ^2^Department of Anatomy, School of Medicine, College of Health Science, Addis Ababa University, Addis Ababa, Ethiopia

## Abstract

**Background:**

Neural tube defects are a type of congenital anomaly caused by an abnormality in the development of the brain and spinal cord during embryogenesis. They cause high rates of mortality, morbidity, and lifelong disability. There are several studies carried out worldwide reporting different findings on the burden and associated factors. The aim of this study is to carry out a systematic review and meta-analysis of the burden of neural tube defects and their associated factors in Africa.

**Methods:**

A total of 58 eligible articles were identified systematically using databases such as PubMed, Embase, African Journal Online Library, ProQuest, Cochrane, Google Scopus, Google Scholar, and Grey literature. Extracted data were analyzed using STATA 16.0 statistical software. The heterogeneity of studies was determined using the Cochrane Q test statistic and *I*^2^ test statistics with forest plots. A random effects model was used to examine the pooled burden of neural tube defects, subgroups of the region, subtypes of NTDs, sensitivity analysis, and publication bias. The association between NTDs and associated factors was studied using a fixed-effect model.

**Results:**

Fifty-eight studies with a total of 7,150,654 participants in 16 African countries revealed that the pooled burden of neural tube defects was 32.95 per 10,000 births (95% CI: 29.77-36.13). The Eastern African region had the highest burden in the subgroup analysis, with 111.13 per 10,000 births (95% CI: 91.85–130.42). South African countries had the lowest burden, at 11.43 per 10,000 births (95% CI: 7.51–15.34). In subtype analysis, spina bifida had the highest pooled burden at 17.01 per 10,000 births (95 percent CI: 15.00-19.00), while encephalocele had the lowest at 1.66 per 10,000 births (95% CI: 1.12-2.20). Maternal folic acid supplementation (AOR: 0.38; 95% CI: 0.16-0.94), alcohol consumption (AOR: 2.54; 95% CI: 1.08-5.96), maternal age (AOR: 3.54; 95% CI: 1.67-7.47), pesticide exposure (AOR: 2.69; 95% CI: 1.62-4.46), X-ray radiation (AOR: 2.67; 95% CI: 1.05-6.78), and history of stillbirth (AOR: 3.18; 95% CI: 1.11-9.12) were significantly associated with NTDs.

**Conclusion:**

The pooled burden of NTDs in Africa was found to be high. Maternal age, alcohol consumption, pesticide and X-ray radiation exposure, history of stillbirth, and folic acid supplementation were significantly associated with NTDs.

## 1. Introduction

Neural tube defects (NTDs) are a type of congenital anomaly caused by an abnormality in the development of the brain and spinal cord during embryogenesis [1], with nearly 300,000 cases worldwide annually [2]. The effects they cause in Africa are substantial. NTDs are the world's second most common birth defect (1 in 1000 live births) [3], with the highest rates in northern China (3.7/1000 live births) and Ireland (1.6 per 1000 live births) [4].

The prevalence and etiology of NTDs vary by population [5]. The rate of occurrence increases from the west to the east coast in the United States, with the Appalachian region having the highest frequency [3, 4, 6]. NTD affects approximately 1-3 per 1000 births in Africa each year [7]. The prevalence of NTD in Ghana is 1.6 per 1000 live births [8].

In Ethiopia, variable outcomes ranging from 30.87 to 42.5 per 1000 births were reported on the prevalence of neural tube defects. Improved maternal health, preconception care, folic acid supplementation, and routine fetal anomaly scanning may all help to detect congenital anomalies earlier in pregnancy. Permanent epidemiological surveillance is required to determine the true prevalence at the national and temporal trend levels [9–11].

## 2. Methods

### 2.1. Search Strategy

An inclusive review and meta-analysis were undertaken on the burden and related factors of NTDs in Africa, using both published and unpublished material, regardless of publication period. The databases used to search for relevant studies were PubMed, Cochrane Library, Embase, AJOL, Google Scopus, ProQuest, Google Scholar, and other sources. The reference lists of each incorporated article were also searched manually to optimize the search strategy. The entire database will be systematically searched from December 1, 2021 to February 15, 2022.

The key terms used for the search were (((“prevalence” [All Fields]) OR (“prevalence” [MeSH Terms]) OR (“burden” [All Fields]) OR (“burden” [MeSH Terms]) OR (“epidemiology” [All Fields]))))))))) “neural tube defects” [All Fields]) OR “birth defects” [All Fields]) OR “congenital malformation” [All Fields]) OR “congenital anomalies” [All Fields])) (additional file 1). The systematic review and meta-analysis were conducted in agreement with the Preferred Reporting Items for Systematic reviews and Meta-Analyses (PRISMA) guideline (additional file 2) [12].

### 2.2. Criteria for Considering Studies

Any study in Africa that met the criteria and included relevant extractable data on the burden and associated factors of NTDs was included.

#### 2.2.1. Study Area

All of the researches were conducted in African countries.

#### 2.2.2. Study Design

This systemic review and meta-analysis comprised of observational studies (cross-sectional, case-control, and cohort) that reported on the burden and associated variables of NTDs.

#### 2.2.3. Language

Both published and unpublished articles in the English language were included.

#### 2.2.4. Population

Studies conducted among newborn babies were considered.

### 2.3. Exclusion Criteria

Irrelevant articles with missing data, duplicate studies, case reports, conference proceedings, and studies in which NTDs were not clearly reported separately excluded after reviewing their full-texts.

### 2.4. Selection of Study

Relevant articles were identified from the aforementioned databases and imported into Mendeley reference manager software X1.19.4 to eliminate duplicate studies. The retrieved studies were also imported into Review Manager Version 5 for evaluation of associated factors. The titles, abstracts, and full-texts of the retrieved articles were independently reviewed by two review authors Reta Wakoya (RW) and Mekbeb Afework (MA). Any disagreements between the reviewer were resolved through discussions.

### 2.5. Methodological Quality Assessment

The quality of relevant articles was assessed using the Joanna Briggs Institute (JBI) critical evaluation tool [13]. Two of the authors (RW and MA) independently evaluated the quality of the full text selected for the meta-analysis. The tool includes ten items for case-control studies, forty for cross-sectional studies, and eight for cohort studies (additional file 3 and 4). Each study's items were scored as yes (1) or no (0). The quality of each study was graded based on the number of items judged “Yes” (1) as low risk < 60%, medium (60-80%), and high >80%.

### 2.6. Data Extraction

Following the inclusion of the relevant papers, the two reviewers (RW and MA) extracted all of the required data separately. To retrieve all qualified articles, a consistent data extraction tool was used with Microsoft Office Excel Software.

For the burden of NTDs, the data extraction tool includes the first author, publication year, country and subregion, study design, setting, sample size, and number of NTDs. The prevalence and number of cases for each of the NTD subtypes were also included. The data for NTD-related factors was extracted in two tables. The pooled odds ratio and its corresponding 95% confidence interval (CI) were calculated based on the original study report (additional file 5 and 6).

### 2.7. Statistical Methods and Analysis

The retrieved data was imported into the STATA/SE version 16 program for all statistical analyses. The heterogeneity of all included studies was assessed using *I*^2^ statistics and the Cochrane Q test. In this meta-analysis, the test revealed significant heterogeneity among included articles (*I*^2^ = 99.4 *percent*, *P* value = 0.00). As a result, a random effects model was used. The Egger test statistics were used to examine the publication bias. The pooled burden and odds ratios, as well as their respective 95% confidence intervals, were displayed using a forest plot. To determine the associated factors for NTDs, the data was entered into Review Manager Version 5, and pooled odds ratios (ORs) with a 95% confidence interval (CI) were calculated.

### 2.8. Operational Definition

NTDs are a circumstance that influences all neonates with one of the following malformations: spina bifida, anencephaly, and encephalocele. The number of infants born with NTDs was divided by the total number of newborns and multiplied by a hundred to calculate the burden of neural tube defects.

Spina bifida is a congenital disorder caused by the spine and spinal cord not developing properly. In infants with spina bifida, a portion of the neural tube does not close or develop normally, leading to abnormalities in the spinal cord and spine bones. There are three types of spina bifida: myelomeningocele, meningocele, and occulta.

Anencephaly is a birth defect in which the baby's brain and skull bones do not fully form while it is inside the womb. As a result, the cerebral cortex in particular does not develop as quickly in the baby's brain.

Encephalocele is a rare type of congenital abnormality of the neural tube that affects the brain. An encephalocele is a sac-like protrusion or extension of the brain and the membranes that protect it through a hole in the skull.

## 3. Results

The numerous subheadings listed below describe the findings from this systematic review and meta-analysis.

### 3.1. Description of Study Selection

A total of 547 studies on the burden of neural tube defects and associated factors in African countries were found in the Medline (PubMed), Cochrane, Embase, AJOL, ProQuest, Google Scopus, Google Scholars, ResearchGate, Basebieled scholarly, Wiley Online Library, and Google databases.

**Figure 1:**
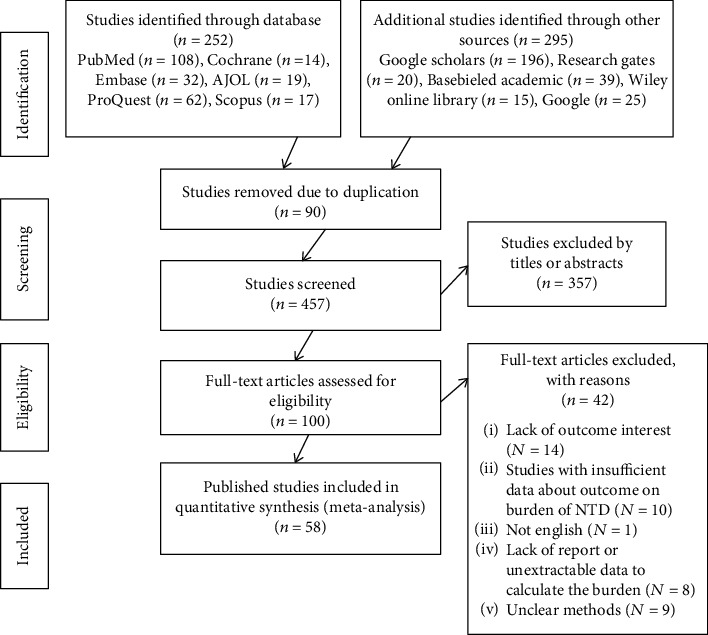
PRISMA flow diagram depicting the selection process of studies for the systematic review and meta-analysis on the burden and associated factors of neural tube defects in Africa, 2022.

Ninety articles were removed due to duplication. After reviewing the titles and abstracts of the remaining 457 papers, it was determined that 375 were irrelevant to the study and were thus eliminated. In addition, 100 full-text papers were assessed for eligibility based on the established criteria. For the final systematic review and meta-analysis, 58 papers were selected that met the criteria (Figure 1).

The overall burden of neural tube defects was determined using 48 studies from among the qualifying papers. Ten more studies were considered to be explored for associated factors of neural tube defects, including ten case-control studies.

### 3.2. Characteristics of the Included Studies

In this systematic review and meta-analysis, 58 studies from sixteen African countries were considered eligible for assessing the prevalence of neural tube defects. Three African countries were included in the analysis of associated factors, with a total sample size of 7,150,654 participants, ranging from 61 in western African countries [14] to 3,803,889 in northern Africa [15]. Eastern African countries accounted for 23 (39.66%) [9–11, 16–34], with Ethiopia having the most 18 (31.03%) [9–11, 16–22, 28–34] and Uganda having two (3.345%) [26, 27]. Kenya, Eritrea, and Tanzania each had one study (1.72%) [23–25].

There were 12 (20.69%) studies [8, 14, 35–44] from western African countries, with Nigeria having the most with 11 (18.97%) [14, 35, 37–45] and Ghana having one (1.72%) [8] research. Eleven (18.97%) research [15, 46–55] was from northern African countries, with three (5.17%) studies [15, 46, 48–51] each from Morocco and Tunisia, two (3.45%) studies [52–55] each from Sudan and Algeria, and one (1.72%) study [47] from Libya. South Africa had the most studies from southern Africa region, with seven (12.06%) [56–62] and Botswana had only one (1.74%) [63]. Three studies (5.17%) [64–66] were from Central African countries, all from the Democratic Republic of the Congo. All research published between 1982 and 2022 that meet the criteria were eligible. There were forty cross-sectional studies (68.97%) [8–10, 14–18, 23, 24, 26, 27, 31–37, 40–44, 47–49, 52, 57–63, 65–68], eight cohort studies (13.79%) [17, 29, 38, 39, 46, 55, 56, 64], and ten case-control studies (17.24%) [19–22, 28, 30, 32, 50, 51, 54] in this analysis. Furthermore, 19 (32.76%) of the studies were rated as high quality. Only three (5.17%) studies were judged low quality, whereas 36 (62.07%) were considered medium quality (Table 1 parts a, b, c, and d and Table 2 parts a and b).

### 3.3. Meta-Analysis

#### 3.3.1. Burden of Neural Tube Defects in Africa

**Figure 2:**
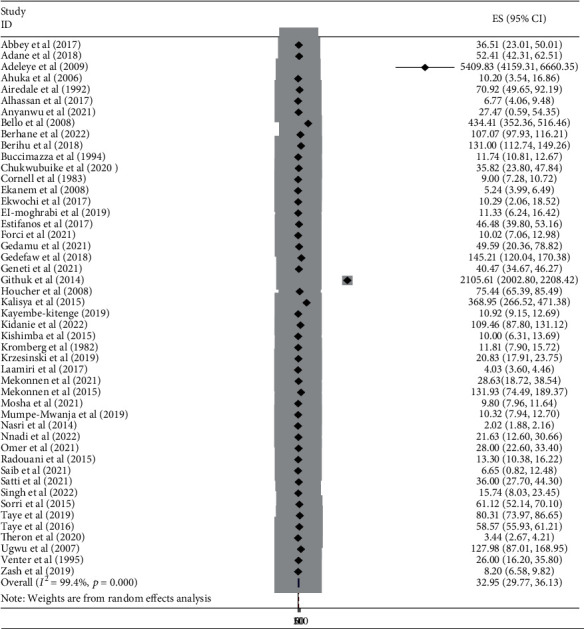
A forest plot depicting the burden of neural tube defects per 10,000 births in Africa, 2022.

In this meta-analysis, to assess the burden of neural tube abnormalities in Africa, 48 studies were found to be included. The overall burden of neural tube defects was 32.95 per 10,000 infants on average (95% CI:29.77-36.13) [8–11, 14–18, 23–27, 29, 31–34, 36–44, 46–49, 52, 53, 55–66, 70, 72]. As a result, the random effects model was used to estimate the prevalence of neural tube defects in African countries (Figure 2).

#### 3.3.2. Publication Bias

**Figure 3:**
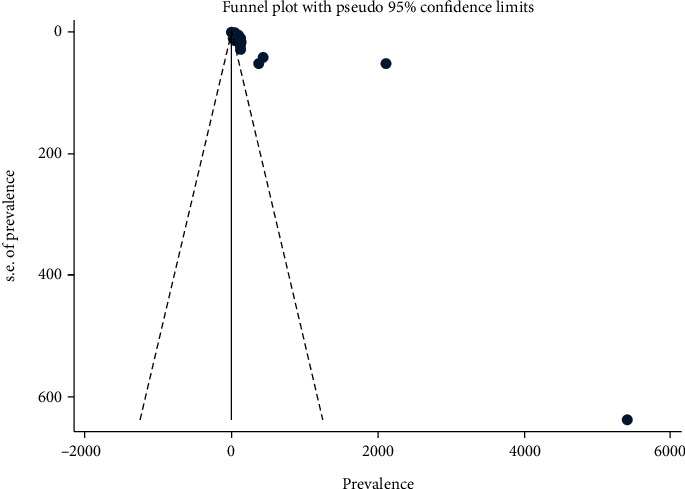
The funnel graph depicts the distribution of studies included in the burden of neural tube defects in Africa, 2022.

As indicated by the asymmetrical distribution of funnel plot tests, there was a publication bias in the burden of neural tube defects among the included studies. The forest plot revealed that the experiments differed greatly. The studies showed significant heterogeneity (*I*^2^ = 99.4%, *P* value ≤ 0.001) (Figure 3). Egger's test, too, revealed that publication bias is statistically significant (*p* value ≤ 0.001) (Table 3).

#### 3.3.3. Sensitivity Analysis for the Burden of Neural Tube Defects in Africa

**Figure 4:**
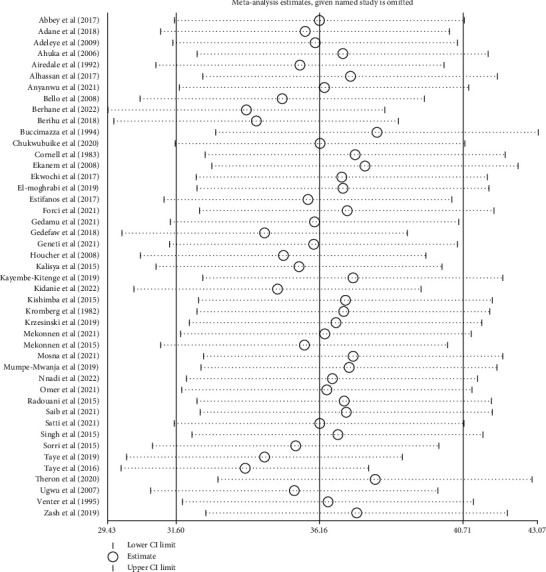
Results of sensitivity analysis of 45 studies after removing the three studies [15, 24, 48] in Africa, 2022.

A sensitivity analysis was utilized to evaluate the influence of various studies on the pooled burden of NTDs in Africa. Except for three studies, it was established that the pooled burden of NTDs was estimated to be uniform across studies. After removing just one study, the burden has been determined to be 37.54 (33.41-41.58) [48]. The burden was 38.56 (34.18-42.95) after removing only one [15]. After removing only one [24], it was 29.91 (27.06-32.76). If the three studies had been excluded together, the overall estimated prevalence would have been 36.16 (31.60-40.71) [15, 24, 48] (Figure 4).

#### 3.3.4. Subgroup Analysis of the Burden of Neural Tube Defects

**Figure 5:**
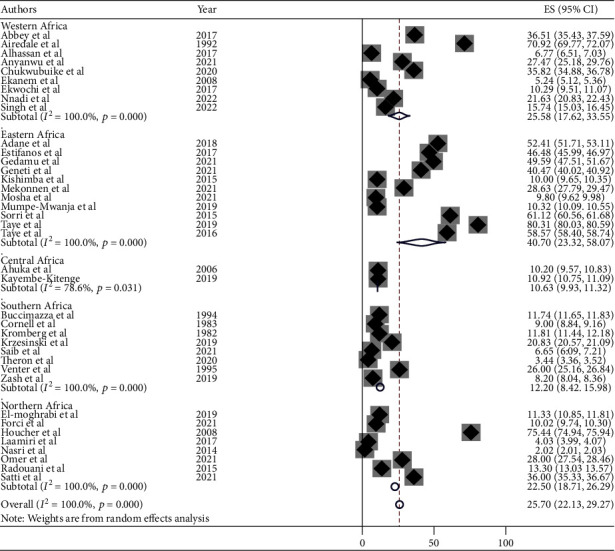
Forest plot of subgroup analysis by the regions of showing the burden of neural tube defects per 10,000 births in Africa, 2022.

Subgroup analysis was carried out based on the regions where the studies were conducted. The highest burden was observed in the eastern Africa region with a burden of 111.13 per 10,000 births (95% CI: 91.85, 130.42) followed by western Africa at 34.39 per 10,000 (95% CI: 23.78, 45.01), Central Africa at 26.66 per 10,000 (95% CI: 3.88, 49.45), and northern Africa at 15.93 per 10,000 (95% CI: 12.96, 18.90). The lowest burden was observed in southern Africa, with 11.43 per 10,000 births (95% CI: 7.51, 15.34) (Figure 5).

#### 3.3.5. Burden of NTDs by Subtypes in Africa

**Figure 6:**
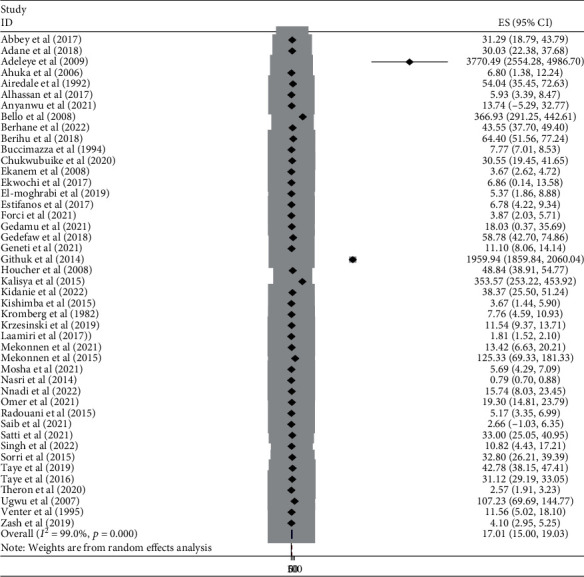
Forest plot showing the burden of spina bifida per 10,000 in Africa, 2022.

**Figure 7:**
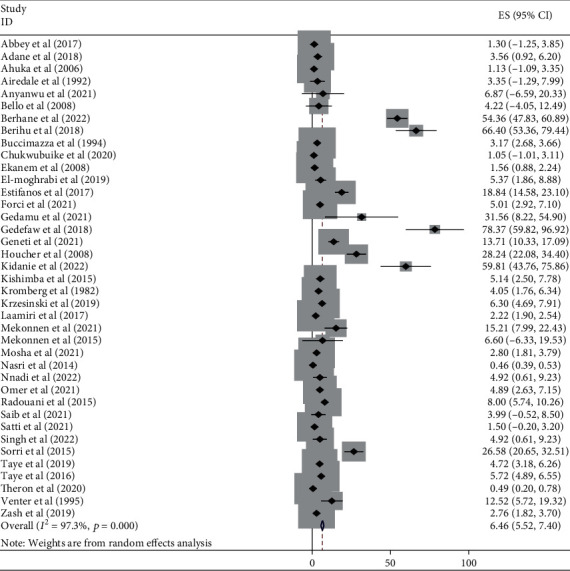
Forest plot depicting the burden of anencephaly per 10,000 in Africa, 2022.

**Figure 8:**
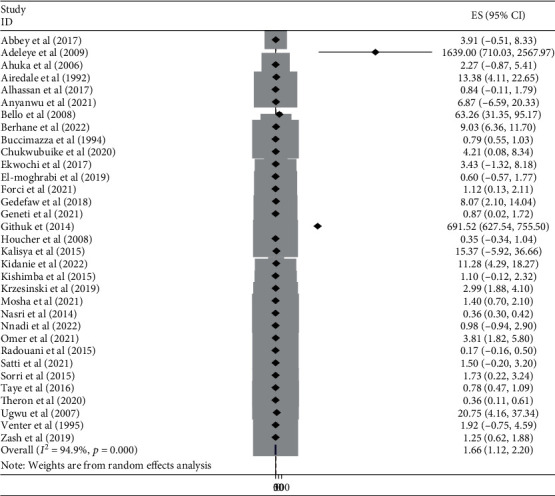
Forest plot showing the burden of encephalocele per 10,000 in Africa, 2022.

**Figure 9:**
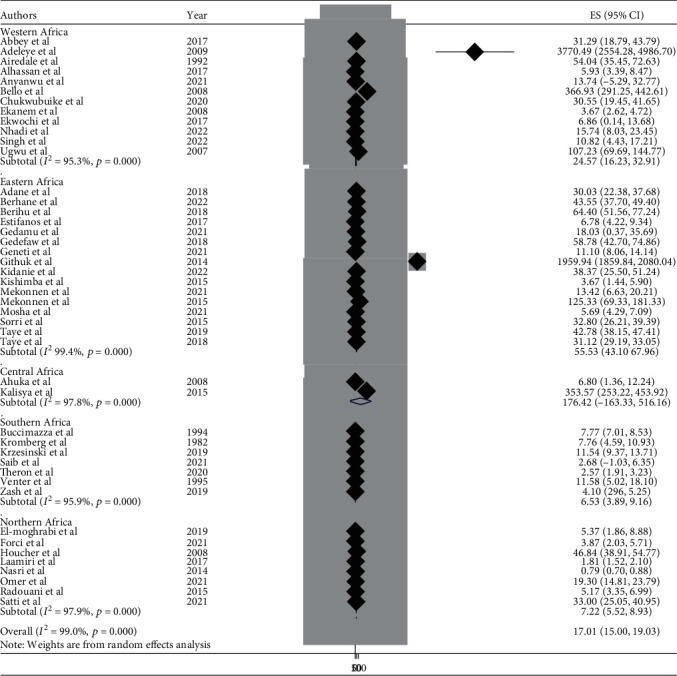
Forest plot showing the burden of spina bifida by subregion per 10,000 in Africa, 2022.

**Figure 10:**
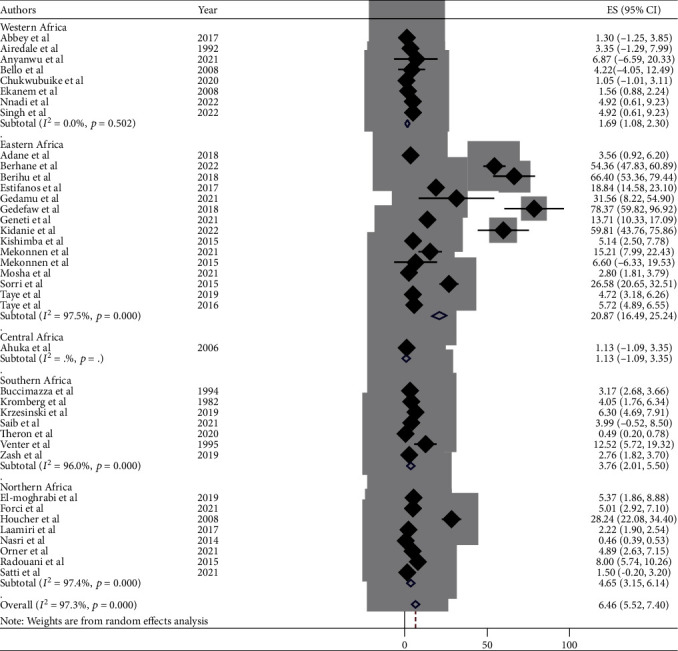
Forest plot depicting the pooled burden of anencephaly by subregion per 10,000 in Africa, 2022.

**Figure 11:**
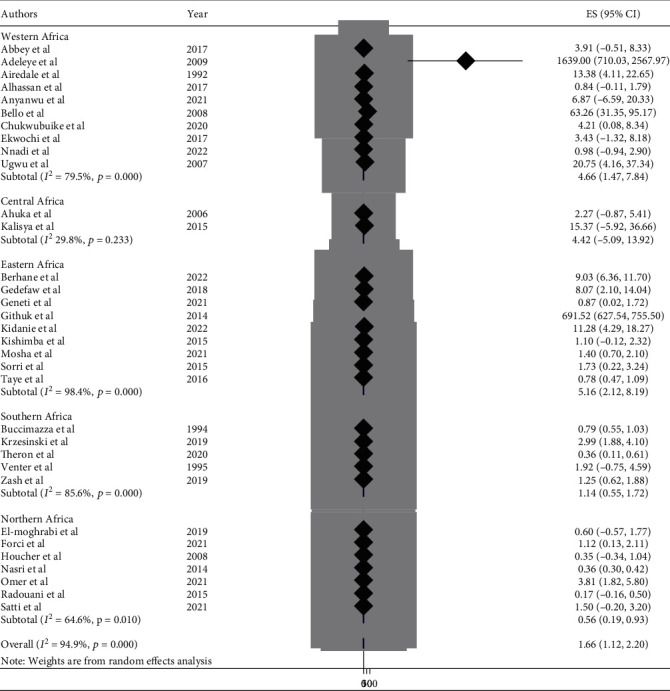
Forest plot showing the pooled burden of encephalocele by subregion per 10,000 in Africa, 2022.

This meta-analysis looked into the subtypes of neural tube defects. Spina bifida had the highest burden among the subtypes of neural tube defects in African countries, at 17.01 (95% CI: 15.00, 19.00) (Figure 6), followed by anencephaly at 6.46 (95% CI: 5.52, 7.40) (Figure 7) and encephalocele at 1.66 (95% CI: 1.12, 2.20) (Figure 8). The burden of spina bifida was highest at 176.42 (95% CI: -163.33, 516.16) in Central Africa and lowest at 6.53 (95% CI: 3.89, 9.16) in southern Africa countries (Figure 9). The burden of anencephaly was highest at 20.87 (95% CI: 16.49, 25.24) in eastern Africa countries and lowest at 1.13 (95% CI: 1.09, 3.35) in Central African countries (Figure 10). The frequency of encephalocele was highest at 5.16 (2.12, 8.19) in Eastern African countries and lowest at 0.56 (0.19, 0.93) in northern African countries (Figure 11).

#### 3.3.6. Associated Factors of Neural Tube Defects

This meta-analysis included maternal folic acid supplementation, parental consanguinity marriage, maternal alcohol consumption, maternal history of stillbirth, maternal pesticide exposure, maternal radiation exposure, maternal medical illness, infant sex, and mother's age > 35 years old as associate factors for neural tube defects, with ten papers [19–22, 28, 30, 32, 51, 54, 71] retrieved into the review. For sensitivity analysis, each variable was carefully examined. However, sensitivity analysis revealed that neither of the linked parameters was significant. A separate analysis was conducted for each variable.

**Figure 12:**
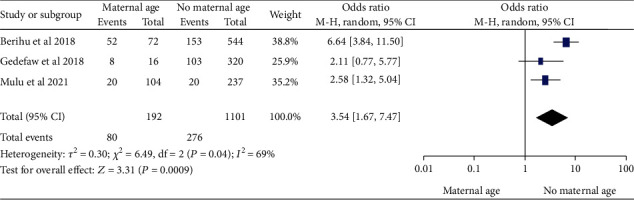
Forest plot showing the association between maternal age and the neural tube defects in Africa, 2022.

*(1) Maternal Age and Neural Tube Defects*. From three [20, 21, 30] studies, we found that maternal age above 35 years of age during pregnancy was significantly associated with NTDs among newborn infants with odds ratio 3.54 (95% CI: 1.67–7.47). This suggests that infants born to mothers over 35 were 3.54 times more likely to have NTDs during pregnancy than infants born to mothers under 35. The study showed moderate heterogeneity (*I*^2^ = 69%, *P* ≤ 0.001) (Figure 12); hence, random model effect was used.

**Figure 13:**
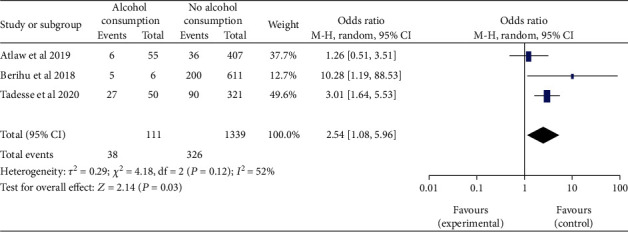
Forest plot depicting the association between maternal alcohol consumption and neural tube defects in Africa, 2022.

*(2) Maternal Alcohol Consumption and Neural Tube Defects*. Three [22, 28, 30] studies showed that the women who had alcohol consumption during pregnancy were significantly associated with NTDs, odds ratio 2.54 (95% CI: 1.08-5.96). This indicated that infants of mother who had alcohol consumption were 2.54 times more likely to have babies with NTDs than women who had never consumed alcohol during pregnancy. The study showed moderate heterogeneity (I^2^ = 52%, P <0.12) (Figure 13). Hence, random model effect was computed.

**Figure 14:**
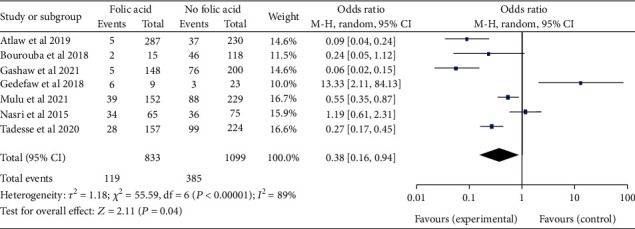
Forest plot illustrating the associations between maternal folic acid supplementation and the burden of neural tube defects in Africa, 2022.

*(3) Maternal Folic Acid Supplementation and Neural Tube Defect*. From seven [20–22, 28, 32, 54, 71] studies, we found that maternal folic acid supplementation during pregnancy was significantly associated with NTDs, odds ratio 0.38 (95% CI: 0.16-0.94). In epidemiological expressions, this showed us that infants born from mothers who took folic acid supplementation during pregnancy were 62% times less likely to have NTDs. The study showed high heterogeneity (*I*^2^ = 89%, *P* ≤ 0.001) (Figure 14). Hence, random model effect was used.

**Figure 15:**
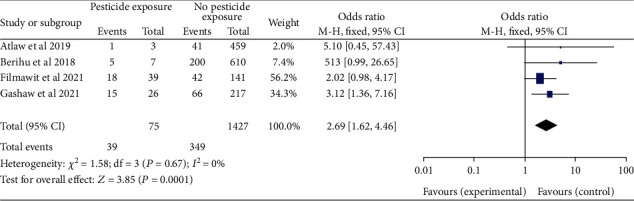
Forest plot showing the linkage between the maternal exposure to pesticide and neural tube defects in Africa, 2022.

*(4) Maternal Exposure to Pesticide and Neural Tube Defects*. This research looked at four studies [19, 20, 28, 30] to assess the maternal exposure to pesticide and neural tube defects. The neural tube defect pooled odds ratio found that women who were exposed to pesticides during pregnancy were 2.69 times more likely to have a baby with neural tube defects (OR, 95 percent CI; 2.69 (1.62-4.46)). The study was considered with the heterogeneity test, which found low heterogeneity (I^2^ = 0, *P* < 0.67) (Figure 15). As a result, the fixed-effects model was adopted in this study.

**Figure 16:**
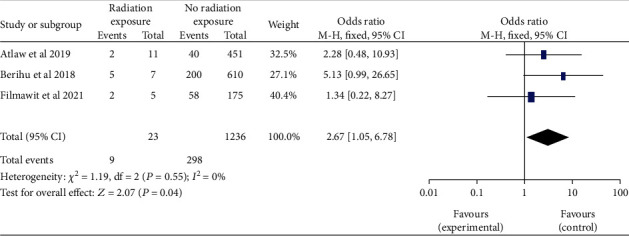
Forest plot showing the linkage between the maternal exposure to X-ray radiation and neural tube defects in Africa, 2022.

*(5) Maternal Exposure to X-Ray Radiation and Neural Tube Defects*. Three studies [19, 28, 30] looked at maternal radiation exposure during pregnancy to realize if there was a link to neural tube defects. The combined odds were analyzed. Women who had been exposed to radiation were 2.67 times more likely to have infants with neural tube defects than women who had not been exposed to radiation (OR, 95% CI: 2.67 (1.05-6.78)). Low heterogeneity (I^2^ = 0, *P* < 0.55) was found. This study utilized a fixed-effects model (Figure 16).

**Figure 17:**
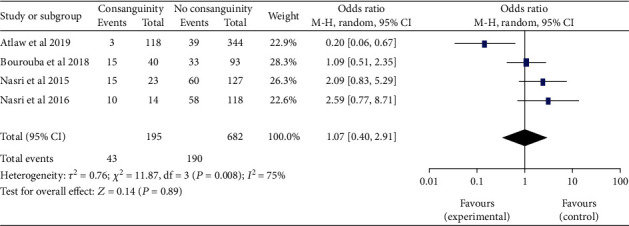
Forest plot depicting the association between the parental consanguineous marriage and neural tube defects in Africa, 2022.

*(6) Paternal Consanguineous Marriage and Neural Tube Defects*. Based on the four studies [28, 51, 54, 71], the link between parental consanguineous marriage and neural tube defects was rigorously analyzed. There was no difference in the combined odds ratio of neural tube defects between parents who were consanguineous and those who were not (OR 95 percent CI: 1.07 (0.40-2.91)). *I*^2^ = 75% and*P* ≤ 0.001revealed moderate heterogeneity. As a result, in the final analysis, the random effects model was used (Figure 17).

**Figure 18:**
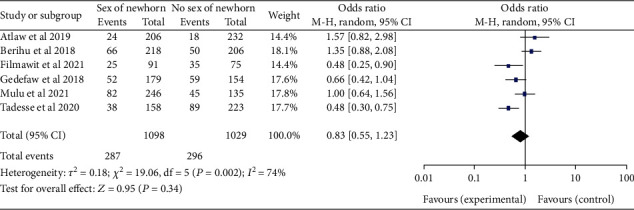
Forest plot illustrating the association of the sex of newborn infant and neural tube defects in Africa, 2022.

*(7) Sex of Newborn Infants and Neural Tube Defects*. The results of the six studies [19, 21, 22, 28, 30, 32] revealed that there is no difference in the burden of neural tube defects between male and female newborns (OR, 95% CI: 0.83 (0.55-1.23)). The heterogeneity was then found to be moderate (*I*^2^ = 74%, *P* ≤ 0.001). The random effect was used (Figure 18).

**Figure 19:**
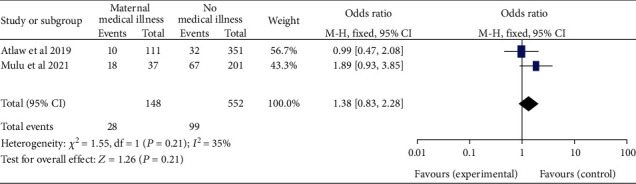
Forest plot showing the association of maternal medical illness and neural tube defects in Africa, 2022.

*(8) Maternal Medical Illness and Neural Tube Defects*. The pooled odds ratio for neural tube defects between the two studies [21, 28] found no difference between pregnant women with and without medical illness (OR, 95 percent CI: 1.38 (0.83-2.28)) (Figure 19). The heterogeneity was then found to be low (*I*^2^ = 35%, *P* < 0.20). The fixed effect was therefore assessed.

**Figure 20:**
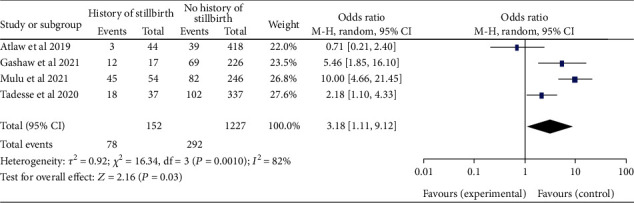
Forest plot depicting the association of the maternal history of stillbirth and neural tube defects in Africa, 2022.

*(9) Maternal History of Stillbirth and the Outcome*. Three [20–22, 28] studies revealed that women who had a stillbirth were 3.18 times more likely to have a newborn with neural tube defects than women who had not had a stillbirth, (95% CI: 1.11–9.12). The study discloses high heterogeneity (*I*^2^ = 82%, *P* ≤ 0.001). As a result, a random effects model was contemplated (Figure 20).

## 4. Discussion

The burden of subtype analysis of neural tube defects was evaluated by region for the first time in Africa. The overall burden of NTDs was 32.95 per 10,000 infants (95% CI: 29.77–36.13). This finding is higher than the studies conducted in California which reported a prevalence of 9 per 10,000 births [73], low- and middle-income countries (11 per 10,000) [74], Africa (21.42 per 10,000) [69]. However, the overall burden found in this study is lower than the previous studies conducted in Africa which reported a prevalence value of 50.74 per 10,000 and India (45 per 10,000 and 42.48 per 10,000) [75–77]. This may be due to several factors, including variable birth record system, study design, and population size. Inclusion or exclusion criteria of articles among reviews may also have an effect in the findings. Furthermore, data loss and less organized system might also be a reason for the heterogeneity of the burden rate among the various studies.

The subgroup analysis of this study showed that the burden of NTDs among newborn infants significantly varies across the regions. The highest burden was observed in the eastern Africa region with a burden of 111.13 per 10,000 births (95% CI: 91.85, 130.42), and the lowest burden was in southern Africa, with 11.43 per 10,000 births (95% CI: 7.51, 15.34). This has been linked to varying folic acid supplementation trends. The eastern region had a higher burden. This may be due to a greater deficiency in folic acid supplementation with the staple foods lacking adequate amount of folic acid fortification. On the other hand, the reason for the lower burden in the southern countries may be due to better folic acid supplementation practices and fortification policies in their staple foods [78–80].

In African countries, spina bifida had the highest burden among the subtypes of NTDs with 17.01 (95% CI: 15.00, 19.00), followed by anencephaly at 6.46 (95% CI: 5.52, 7.40) and encephalocele at 1.66 (95% CI: 1.12, 2.20). Based on the regions, the burden of spina bifida was highest at 176.42 (95% CI: -163.33, 516.16) in central Africa and lowest at 6.53 (95% CI: 3.89, 9.16) in southern African countries. The burden of anencephaly was highest at 20.87 (95% CI: 16.49, 25.24) in eastern African countries and lowest at 1.13 (95% CI: 1.09, 3.35) in central African countries. The burden of encephalocele was highest at 5.16 (2.12, 8.19) in eastern African countries and lowest at 0.56 (0.19, 0.93) in northern African countries. The current review showed that the burden of spina bifida was higher than a previous study conducted in Africa with a value of 13 per 10,000 births [81]. On the other hand the burden of anencephaly and encephalocele were lower than that of a previous study carried out in Africa, with a value of 14 per 10,000 and 2 per 10,000, respectively [82, 83]. This variation in the burden of the subtypes of NTD may be due to the differences in defect identification and detection methods over time, as well as differences in location, income level, and institutional folic acid fortification policy [74, 80, 84].

In this study, 10 studies [19–22, 28, 30, 32, 51, 54, 71] were retrieved in order to identify protective and associated factors with NTDs in newborns. After reviewing 2,946 sample sizes, 946 cases and 1703 controls were investigated. Infants born to mothers over the age of 35 during pregnancy were 3.54 times more likely to have NTDs than infants born to mothers under the age of 35, according to the combined odds. A finding of this study, which a fetus with NTDs is more likely to have chromosomal abnormalities as maternal age increases, was consistent with a previous study that found a link between maternal age and infants born with NTDs that have chromosomal abnormalities [85]. Meiotic division restriction leads to increased aneuploidy [86, 87].

Women with a history of alcohol use were 2.54 more likely to have infants with NTDs than women that had never consumed alcohol during pregnancy. This finding contradicts with the findings from studies that reported no link between maternal alcohol consumption and NTDs. This finding in the study has explained very well that this could be due to underreporting of alcohol consumption, which is directly related to the social stigma of drinking during pregnancy [88, 89].

Women who had been exposed to radiation were 2.67 times more likely to have infants with neural tube defects than women who had not been exposed to radiation. This finding is in line with the study's findings of altered chromatin structure, altered gene expression, and DNA damage. It may be due to radiation's ability to alter global DNA methylation, which in turn can affect altered gene expression, altered chromatin structure, and DNA damage [90]. Apoptotic mutations caused by X-rays combine with neurodevelopment to increase the likelihood of NTD closure failure. Chronic low-dose radiation may affect the growth of neural progenitor cells and disrupt brain development [90].

The link between parental consanguineous marriage and NTDs has been extensively examined. There was no difference in the combined odds ratio of NTD among with and without consanguineous marriages. This observational study contradicts the findings of an Indian study that had reported that consanguinity increases the risk of NTD [91]. Consanguineous marriage alters the communal genetic frequency due to the gene residing in the family's genetic tree, which has a significant impact on NTD [92].

The results of the present study show no difference between male and female newborns in the burden of neural tube defects. These results go against a study [76] from India that found that newborn girls are more likely than newborn boys to have NTD.

It is unlikely that the outcome can be used to predict vulnerability to NTDs and was most likely caused by the rate of embryonic development. Due to females' slower rate of growth and the length of time spent undergoing neurulation, males appear to have made advancements in a few areas of the neurulation process, which increases the risk of NTDs [93]. The prevalence of NTDs was reduced due to extensive folic acid supplementation in both sexes [94].

The pooled odds ratio for neural tube defects among the studies found no difference between pregnant women with and without medical illness, according to this meta-analysis. These findings are consistent with a study carried out in the United States [95], which found no difference in NTD prevalence between HIV-exposed pregnant women and the general population. On the other hand, it contradicts a previous study that found a higher risk of NTDs linked to fever during the first trimester of pregnancy [96]. In addition, a study done on Mexican-Americans had also reported that the occurrence of stressful life events was associated with NTD risk [97].

Women who had a history of stillbirth were 3.18 times more likely to have a newborn with neural tube defects than women who had not had a stillbirth. This finding is consistent with previous study that a previous miscarriage increases the risk of NTDs. The risk of recurrent stillbirth and termination pregnancies rises with NTD [98].

Reviewing the overall prevalence and contributing factors of neural tube defects among newborns in Africa has revealed significant advancements made possible by incorporating newer studies and up-to-date research to attract more attention and address a variety of studies. Therefore, the main aim of this systematic review and meta-analysis was to estimate the burden of neural tube defects and their associated factors in Africa. In order to minimize the occurrence of neural tube defects in Africa, policymakers and program planners could use the findings of this review as a guide when developing suitable interventions. The evaluation could also be used as a starting point for future studies on associated subjects.

## 5. Strength and Limitations of the Study

Strength of the study is inclusion of cross-sectional, cohort, and case-control type of additional and current studies to increase attention and address many studies to show the burden of NTDs in Africa. On the other hand, as a limitation, only a few original studies have been conducted in some African regions and included in this review. The studies reviewed are hospital-based studies, which may likely limit the coverage of the study population to assess the overall burden of NTDs in Africa.

## 6. Conclusion

This review has revealed the burden of NTDs among newborn infants in African countries. The burden of NTDs varies in different regions of Africa and varies in relation with different risk factors. Folic acid supplementation is found as a protective factor of NTDs. The recommendation of this study is to implement a preconception folic acid therapy policy for all women of childbearing age, as well as a policy of mandatory food fortification is critical.

## Figures and Tables

**(a):** 

Authors	Year	Country	Region	Study design	Setting	Sample size	Number of cases	Burden/10,000	Duration of study/months	Quality of study
Abbey et al. [37]	2017	Nigeria	Western Africa	Cross-sectional	Hospital-based	7,670	28	36.51	48	Medium
Adane and Seyoum [11]	2018	Ethiopia	Eastern Africa	Cross-sectional	Hospital-based	19,650	103	52.41	36	Medium
Adeleye and Olowookere [14]	2009	Nigeria	Western Africa	Cross-sectional	Hospital-based	61	33	5409.83	12	Medium
Garcon and Danielle [65]	2006	DR Congo	Central Africa	Cross-sectional	Hospital-based	8824	9	10.2	96	Medium
Airede [38]	1992	Nigeria	Western Africa	Prospective	Hospital-based	5,977	42	70.92	36	Medium
Alhassan et al. [8]	2017	Ghana	Western Africa	Cross-sectional	Hospital-based	35,426	24	6.77	48	Medium
Anyanwu et al. [39]	2021	Nigeria	Western Africa	Prospective	Hospital-based	1,456	4	27.47	9	Low
Bello et al. [40]	2008	Nigeria	Western Africa	Cross-sectional	Hospital-based	2,371	103	434.41	48	Medium
Berhane and Belachew [29]	2022	Ethiopia	Eastern Africa	Prospective	Hospital-based	48,750	522	107.07	36	High
Berihu et al. [31]	2018	Ethiopia	Eastern Africa	Cross-sectional	Hospital-based	14,903	195	131	9	High
Buccimazza et al. [57]	1994	South Africa	Southern Africa	Cross-sectional	Hospital-based	516,252	606	11.74	240	Medium
Chukwubuike et al. [41]	2020	Nigeria	Western Africa	Cross-sectional	Hospital-based	9492	34	35.82	4	Medium
Cornell et al. [56]	1983	South Africa	Southern Africa	Prospective	Hospital-based	116859	105	9	60	Medium
Ekanem et al. [42]	2008	Nigeria	Western Africa	Cross-sectional	Hospital-based	127929	67	5.24	276	High
Ekwochi et al. [43]	2017	Nigeria	Western Africa	Cross-sectional	Hospital-based	5,830	6	10.29	48	Medium

**(b):** 

Authors	Year	Country	Region	Study design	Setting	Sample size	Number of cases	Burden/10,000	Duration of study/months	Quality of study
El-moghrabi [47]	2019	Libya	Northern Africa	Cross-sectional	Hospital-based	16,765	19	11.33	12	Medium
Estifanos [23]	2017	Eriteria	Eastern Africa	Cross-sectional	Hospital-based	39,803	185	46.48	56	Medium
Forci et al. [46]	2021	Morocco	Northern Africa	Prospective	Hospital-based	43923	44	10.02	65	High
Gedamu et al. [33]	2021	Ethiopia	Eastern Africa	Cross-sectional	Hospital-based	2,218	11	49.59	6	Medium
Gedefaw et al. [32]	2018	Ethiopia	Eastern Africa	Cross-sectional	Hospital-based	8,677	126	145.21	7	Medium
Geneti et al. [9]	2021	Ethiopia	Eastern Africa	Cross-sectional	Hospital-based	45951	186	40.47	60	High
Githuku et al. [24]	2014	Kenya	Eastern Africa	Cross-sectional	Hospital-based	6041	1272	2105.61	72	High
Houcher et al. [55]	2008	Algeria	Northern Africa	Prospective	Hospital-based	28500	215	75.44	36	Medium
Kalisya et al. [64]	2015	DR Congo	Central Africa	Prospective	Hospital-based	1,301	48	368.95	156	Medium
Kayembe-Kitenge et al. [66]	2019	DR Congo	Central Africa	Cross-sectional	Hospital-based	133662	146	10.92	24	High
Kindie and Mulu [34]	2022	Ethiopia	Eastern Africa	Cross-sectional	Hospital-based	8,862	97	109.46	24	Medium
Kishimba et al. [25]	2015	Tanzania	Eastern Africa	Cross-sectional	Hospital-based	28,217	27	10	5	High
Kromberg and Jenkins [58]	1982	South Africa	Southern Africa	Cross-sectional	Hospital-based	29,633	35	11.81	12	Medium
Krzesinski et al. [59]	2019	South Africa	Southern Africa	Cross-sectional	Hospital-based	93609	195	20.83	72	Low
Laamiri [48]	2017	Morocco	Northern Africa	Cross-sectional	Hospital-based	819,224	330	4.03	36	Medium

**(c):** 

Authors	Year	Country	Region	Study design	Setting	Sample size	Number of cases	Burden/10000	Duration of study/months	Quality of study
Mekonnen et al. [16]	2021	Ethiopia	Eastern Africa	Cross-sectional	Hospital-based	11177	32	28.63	72	Medium
Mekonen et al. [17]	2015	Ethiopia	Eastern Africa	Prospective	Hospital-based	1,516	20	131.93	6	High
Barlow-Mosha et al. [26]	2021	Uganda	Eastern Africa	Cross-sectional	Hospital-based	110,752	109	9.8	40	High
Mumpe-Mwanja et al. [27]	2019	Uganda	Eastern Africa	Cross-sectional	Hospital-based	69,766	72	10.32	28	Medium
Nasri et al. [15]	2014	Tunisia	Northern Africa	Cross-sectional	Hospital-based	3,803,889	769	2.02	240	Medium
Nnadi and Singh [43, 44]	2022	Nigeria	Western Africa	Cross-sectional	Hospital-based	10,163	22	21.63	36	High
Oumer et al. [69]	2021	Sudan	Northern Africa	Cross-sectional	Hospital-based	36,785	103	28	12	Medium
Radouani et al. [49]	2015	Morocco	Northern Africa	Cross-sectional	Hospital-based	60,017	80	13.3	48	Low
Saib et al. [60]	2021	South Africa	Southern Africa	Cross-sectional	Hospital-based	7,516	5	6.65	12	Medium
Perinatal [53]	2021	Sudan	Northern Africa	Cross-sectional	Hospital-based	20,000	72	36	5	High
Singh et al. [35]	2015	Nageria	Western Africa	Cross-sectional	Hospital-based	10,163	16	15.74	36	High
Sorri and Mesfin [18]	2015	Ethiopia	Eastern Africa	Cross-sectional	Hospital-based	28,961	177	61.12	36	Medium
Taye et al. [70]	2019	Ethiopia	Eastern Africa	Cross-sectional	Hospital-based	76,201	612	80.31	6	High
Taye et al. [10]	2016	Ethiopia	Eastern Africa	Cross-sectional	Hospital-based	319,776	1873	58.57	48	High

**(d):** 

Authors	Year	Country	Region	Study design	Setting	Sample size	Number of cases	Burden/10000	Duration of study/months	Quality of study
Theron et al. [61]	2020	South Africa	Southern Africa	Cross-sectional	Hospital-based	223,388	77	3.44	60	Medium
Ugwu et al. [36]	2007	Nigeria	Western Africa	Cross-sectional	Hospital-based	2891	31	127.98	37	Medium
Venter et al. [62]	1995	South Africa	Southern Africa	Cross-sectional	Hospital-based	10380	27	26	42	High
Zash et al. [63]	2019	Botswana	Southern Africa	Cross-sectional	Hospital-based	119477	98	8.2	55	Medium

**Table 2:** A descriptive review of studies that reported associated factors for the burden of neural tube defects in Africa, 2022.

Authors	Year	Country	Region	Total size	Case	Control	Variables of associate factors	OR 95% (CI)	Study of quality
Atlaw et al. [28]	2019	Ethiopia	Oromiya-Bale	462	42	420	Maternal folic acid supplementation Parental consanguinity marriage Maternal alcohol consumption Maternal history of stillbirth Maternal exposure to pesticide Maternal exposure to radiation Maternal medical illness Sex of newborn	0.115 (0.044-0.298) 4.9 (1.49-16.17) 0.79 (0.32-1.98) 1.4 (0.416-4.75) 0.196 (0.02-2.21) 0.44 (0.09-2.08) 1.01 (0.48-2.13) 0.72 (0.38-1.37)	Medium
Berihu et al. [30]	2018	Ethiopia	Tigray	617	205	115	Maternal alcohol consumption Maternal exposure to pesticide Maternal exposure to radiation Sex of newborn Maternal age > 35 years	10.28 (1.19-88.5) 5 (0.15-166.6) 5 (0.15-166.6) 0.51 (0.34-0.76) 2.46 (1.33-4.53)	High
Bourouba et al. [54]	2018	Algeria	Banta	48	44	4	Maternal folic acid supplementation Parental consanguinity marriage Maternal age > 35 years	1.17 (0.39-3.49) 0.92 (0.42-1.97) 4.15 (0.89-19.25)	Medium
Aynalem Tesfay et al. [19]	2021	Ethiopia	Addis Ababa	180	60	120	Maternal alcohol consumption Maternal exposure to pesticide Maternal exposure to radiation Sex of newborn	0.57 (0.20-1.45) 2.01 (0.98-4.16) 1.34 (0.23-8.30) 1.70 (0.91-3.20)	Medium
Gashaw et al. [20]	2021	Ethiopia	Amhara-North Shewa	243	81	162	Maternal folic acid supplementation Maternal exposure to pesticide Maternal history of stillbirth	0.16 (0.07-0.33) 5.34 (1.77-16.05) 3.63 (1.03-12.2)	High
Gedefaw et al. [32]	2018	Ethiopia	Addis Ababa	333	111	222	Maternal folic acid supplementation Sex of newborn Maternal age > 35 years	0.47 (0.23-0.95) 0.56 (0.33-0.94)	Medium
Study et al. [21]	2022	Ethiopia	Amhara region	381	127	254	Maternal folic acid supplementation Maternal history of stillbirth Sex of newborn Maternal medical illness Maternal age > 35 years	1.80 (1.15-2.83) 1 (0.63-1.4) 1 (0.33-2.2) 2.04 (1.03-3.4)	Medium
Nasri et al. [71]	2015	Tunisia	Wassila-Bourguiba center	150	75	75	Maternal folic acid supplementation Parental consanguinity marriage	6.75 (1.95-22.07) 10.70 (3.30-22.66)	Medium
Nasri et al. [51]	2016	Tunisia	Wassila-Bourguiba center	132	68	64	Parental consanguinity marriage	2.60 (12.0-0.70)	Medium
Tadesse et al. [22]	2020	Ethiopia	Amhara region	400	133	267	Maternal folic acid supplementation Maternal alcohol consumption Sex of newborn Maternal history of stillbirth	0.27 (0.17-0.44) 0.59 (0.33-1.05) 2.09 (1.34-3.29) 0.61 (0.28-1.29)	High

**Table 3:** Egger's test for detection of publication bias for studies included in the burden of neural tube defects in Africa, 2022.

Egger's test					
Std_Eff	Coef.	Std. err.	*t*	*P* > |*t*|	(95% Conf. interval)
Slope 2.610286	1.407143	.5977176	2.35	0.023	.2040002
Bias 12.44599	9.80027	1.314385	7.46	≤0.001	7.154551

## Data Availability

The datasets used and/or evaluated in this study are available from the corresponding author upon reasonable request.

## Abbreviations

NTDs: Neural tube defects
CI: Confidence interval
JBI: Joanna Briggs Institute
MeSH: Medical subject headings.
